# Supplementary material for: Development of core-collections for Guizhou tea genetic resources and GWAS of leaf size using SNP developed by genotyping-by-sequencing
Source: PeerJ. 2020 Mar 13;8:e8572. doi: 10.7717/peerj.8572 (PMC7075365; doi:10.7717/peerj.8572)
Supplement: Figure S1 [file peerj-08-8572-s001.pdf]

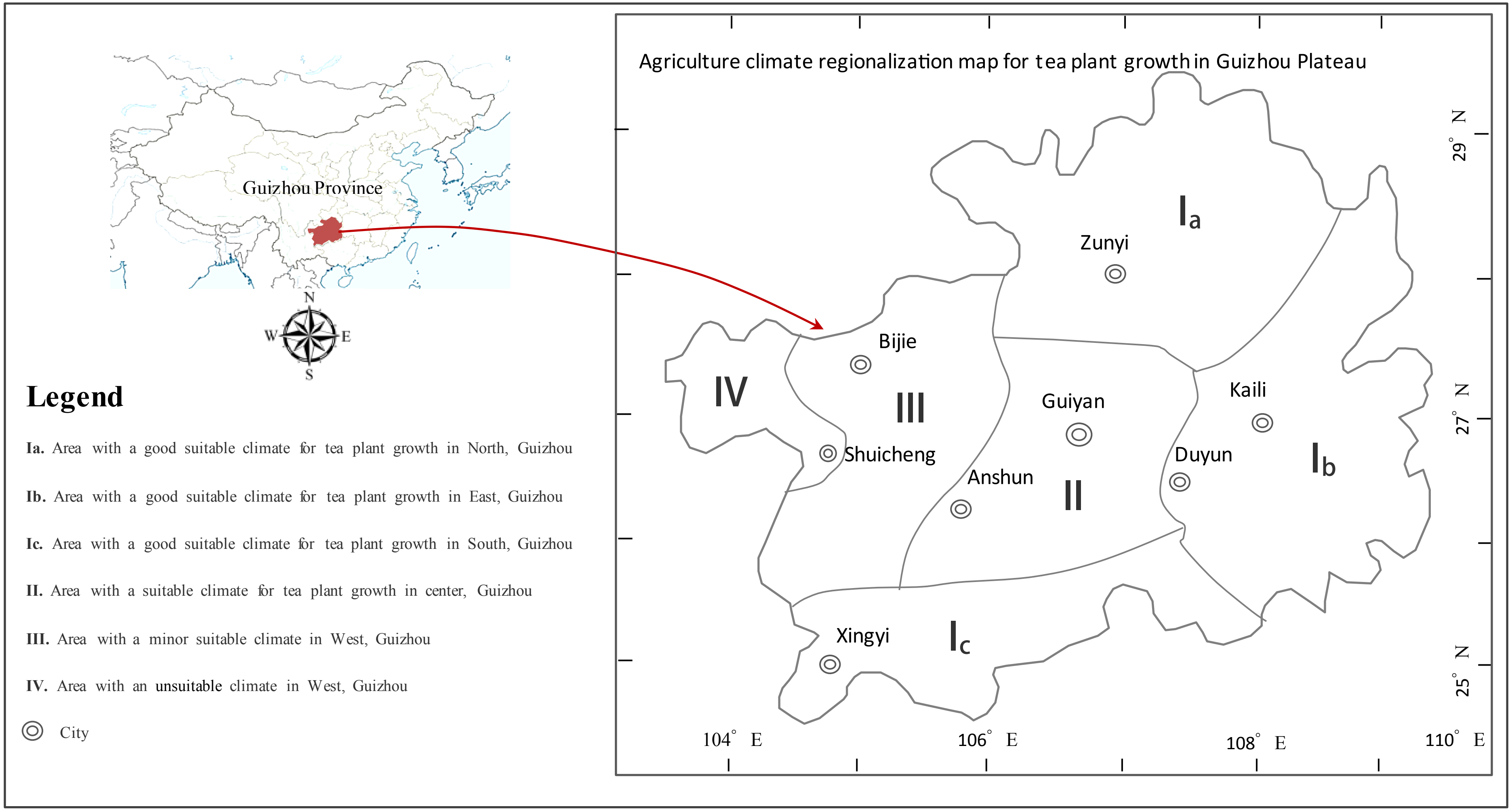
Guizhou Province
N
W
E
S
Legend
Ia. Area with a good suitable climate for tea plant growth in North, Guizhou
Ib. Area with a good suitable climate for tea plant growth in East, Guizhou
Ic. Area with a good suitable climate for tea plant growth in South, Guizhou
II. Area with a suitable climate for tea plant growth in center, Guizhou
III. Area with a minor suitable climate in West, Guizhou
IV. Area with an unsuitable climate in West, Guizhou
City
Agriculture climate regionalization map for tea plant growth in Guizhou Plateau
Ia
Zunyi
Bijie
IV
III
Guiyan
Kaili
Shuicheng
Anshun
II
Duyun
Ib
Xingyi
Ic
104° E
106° E
108° E
110° E
29° N
27° N
25° N
