# Supplementary figures and images for: Development of core-collections for Guizhou tea genetic resources and GWAS of leaf size using SNP developed by genotyping-by-sequencing
Source: PeerJ. 2020 Mar 13;8:e8572. doi: 10.7717/peerj.8572 (PMC7075365; doi:10.7717/peerj.8572)

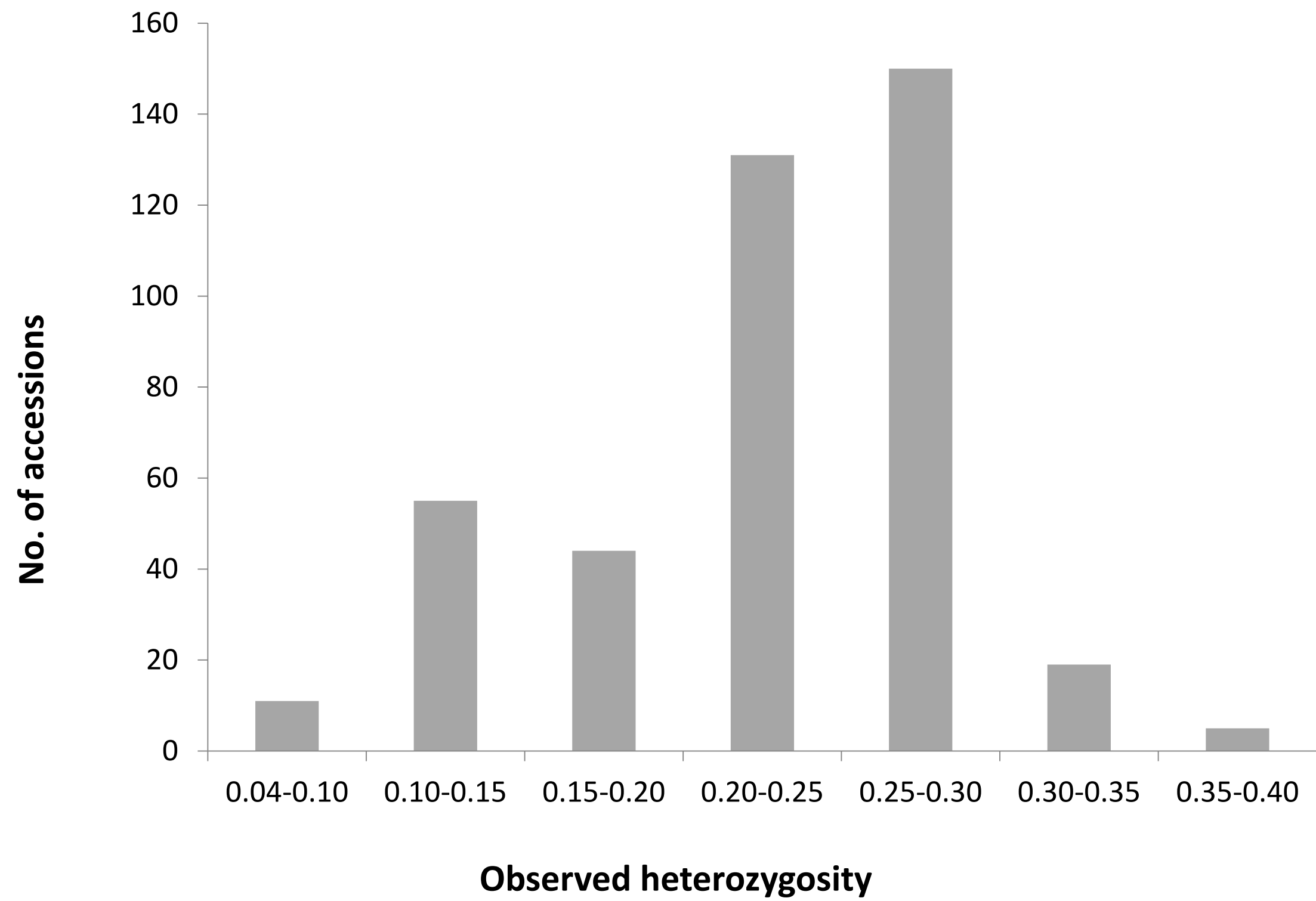

Supplement: Figure S2 [file peerj-08-8572-s002.pdf]

A

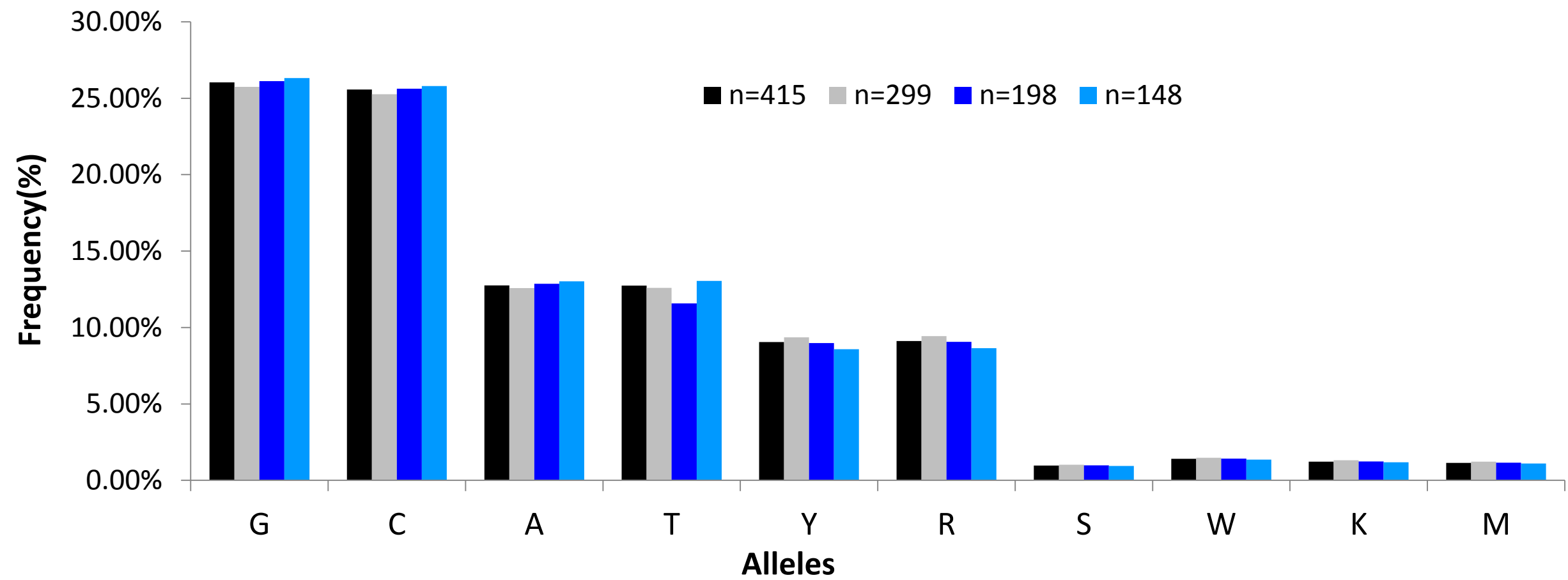

B

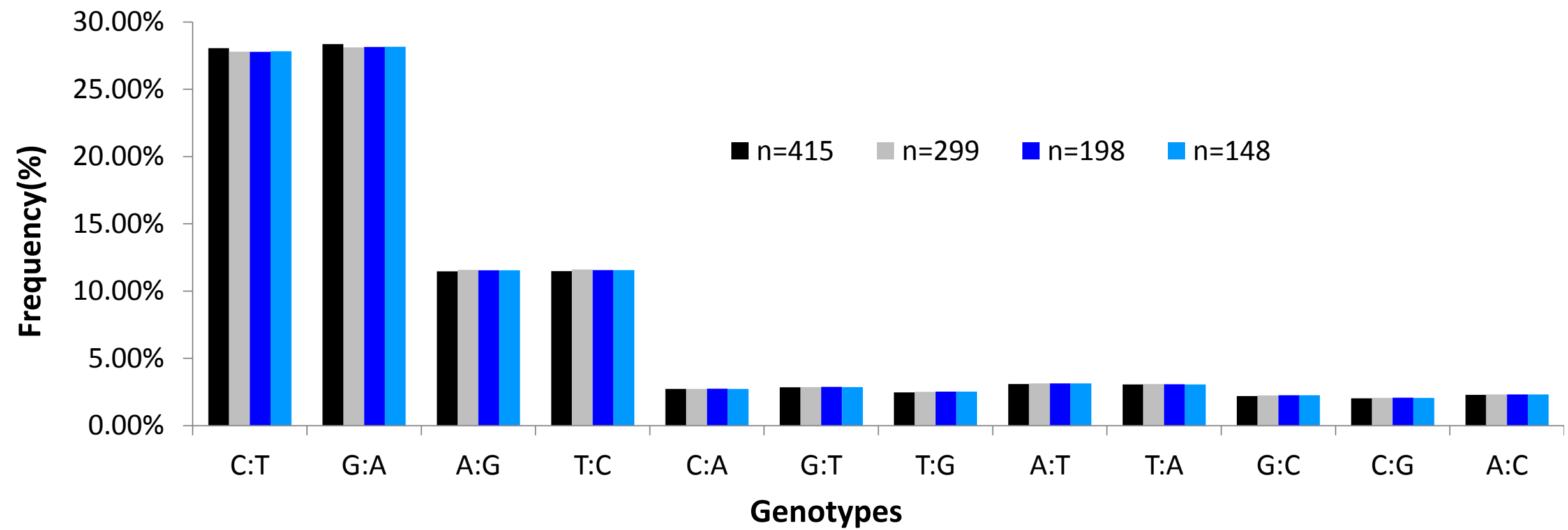

Supplement: Figure S3 — Nucleotide codes are as follows: A, adenine; C, cytosine; G, guanine; T, thymine; Y, C or T; R, A or G; K, G or T; M, A or C; W, A or T; S, G or C (http://www.bioinformatics.org/sms/iupac.html; accessed in Sept. 2017). [file peerj-08-8572-s003.pdf]

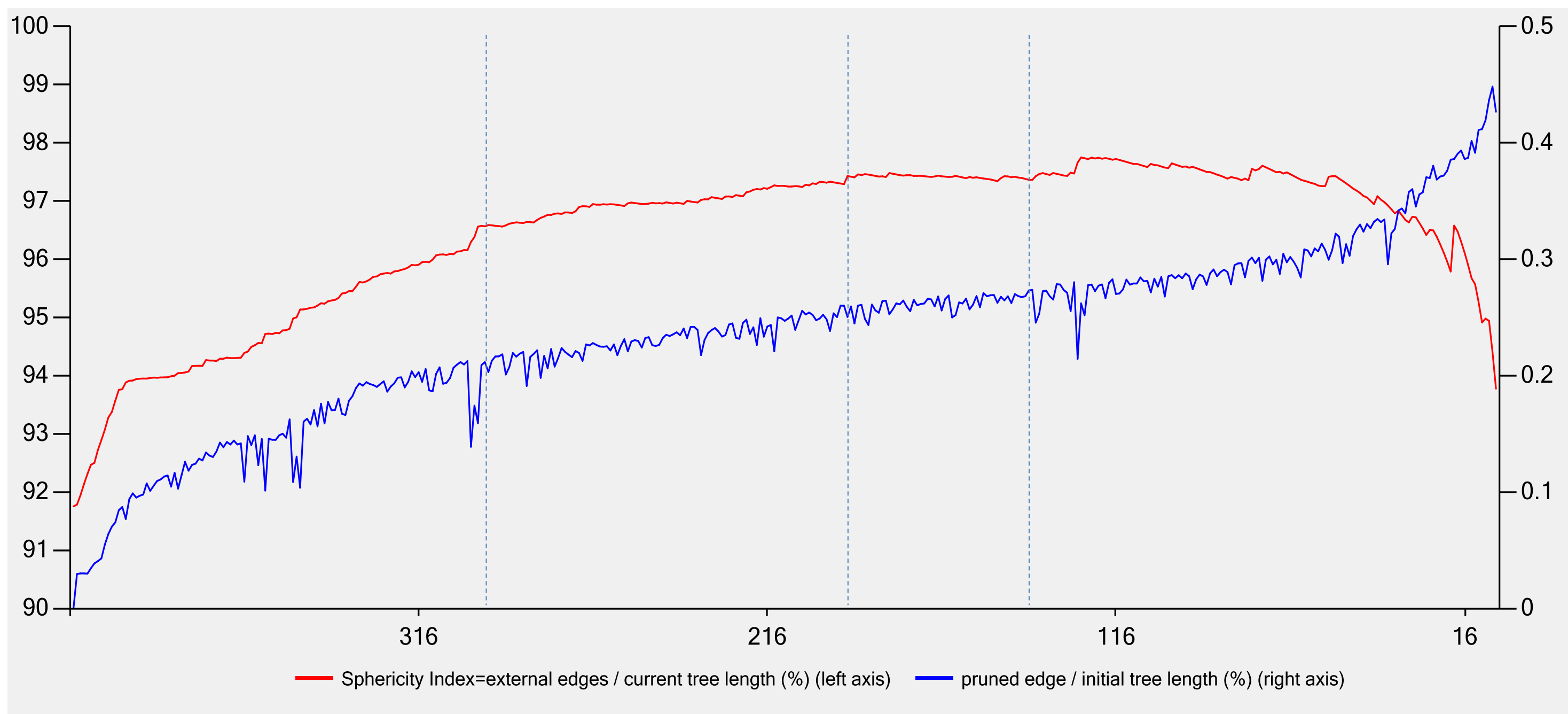

Supplement: Figure S4 [file peerj-08-8572-s004.pdf]

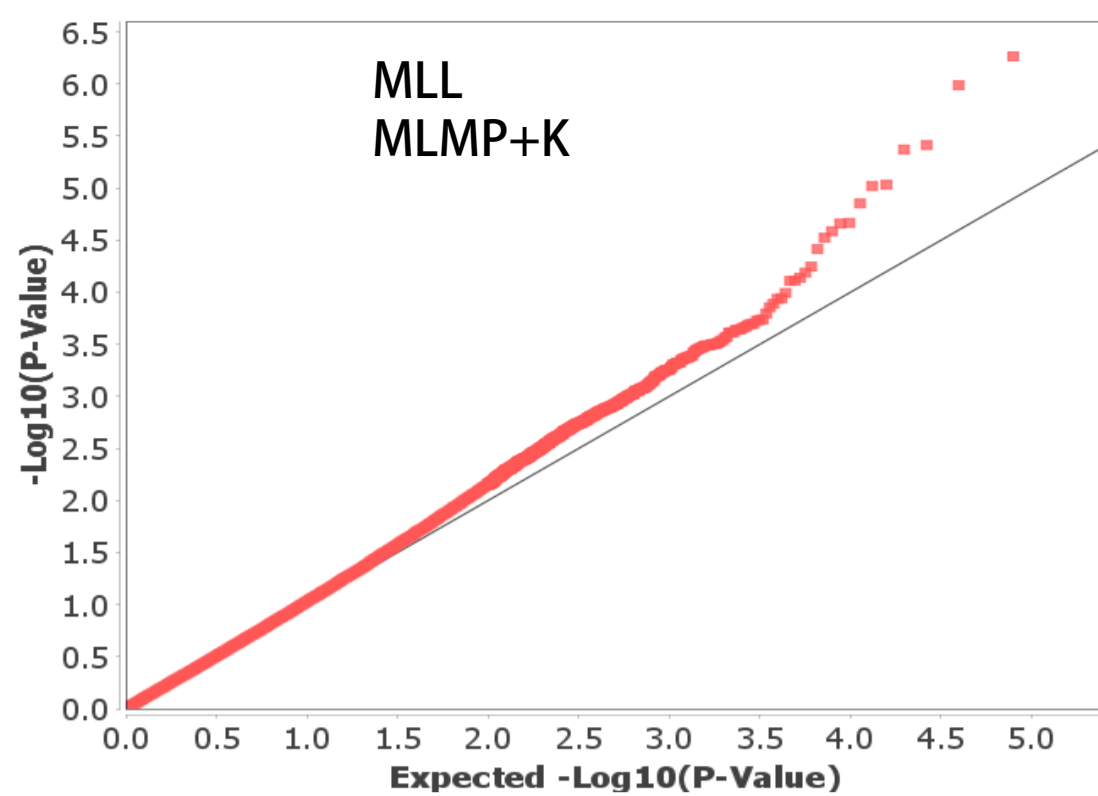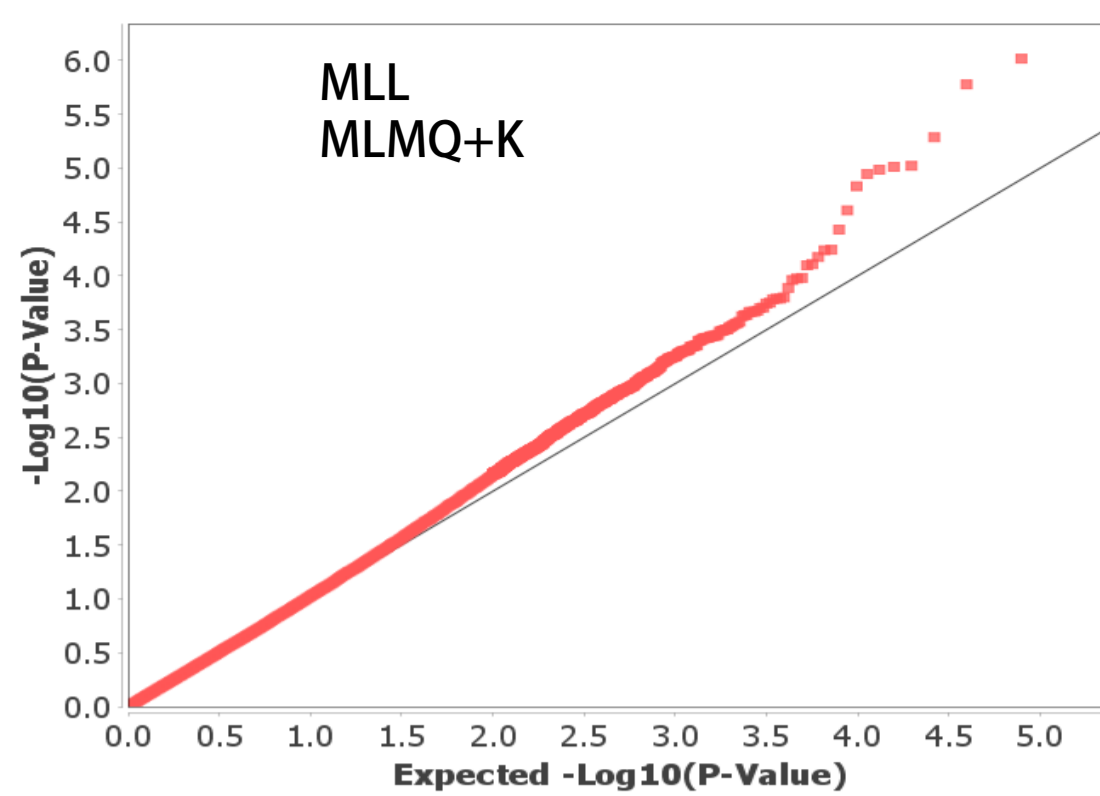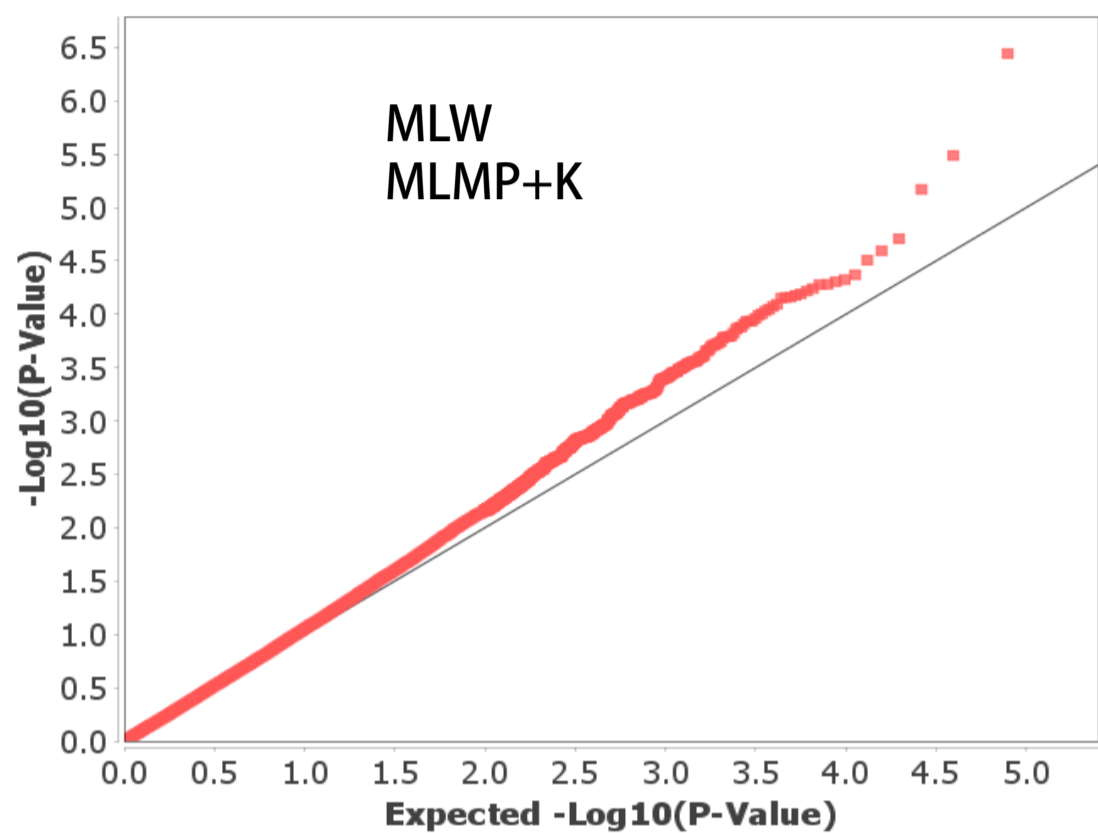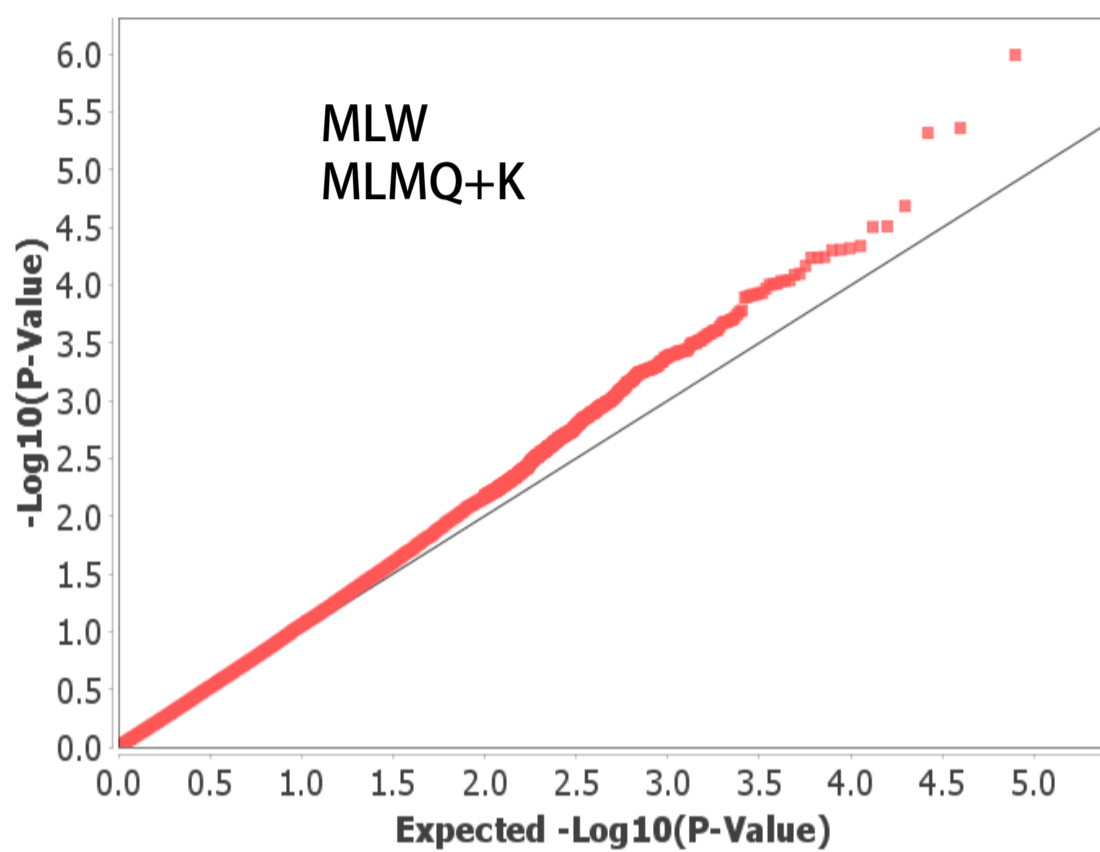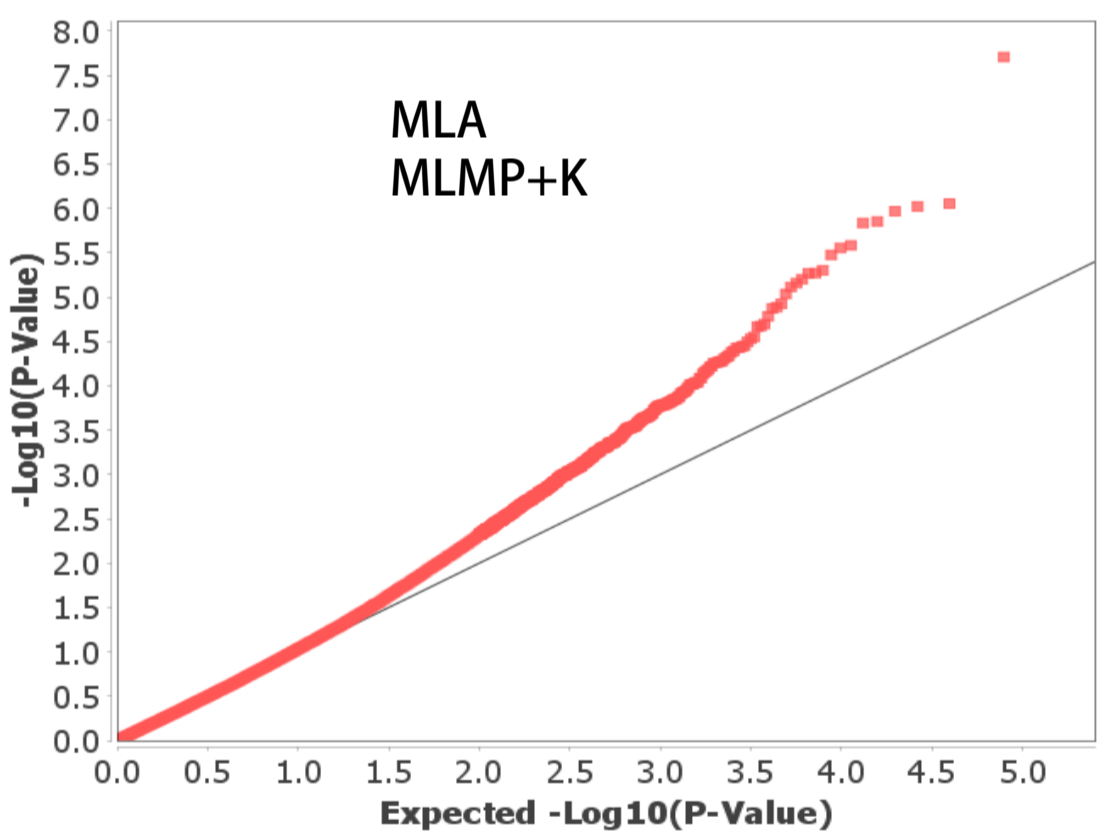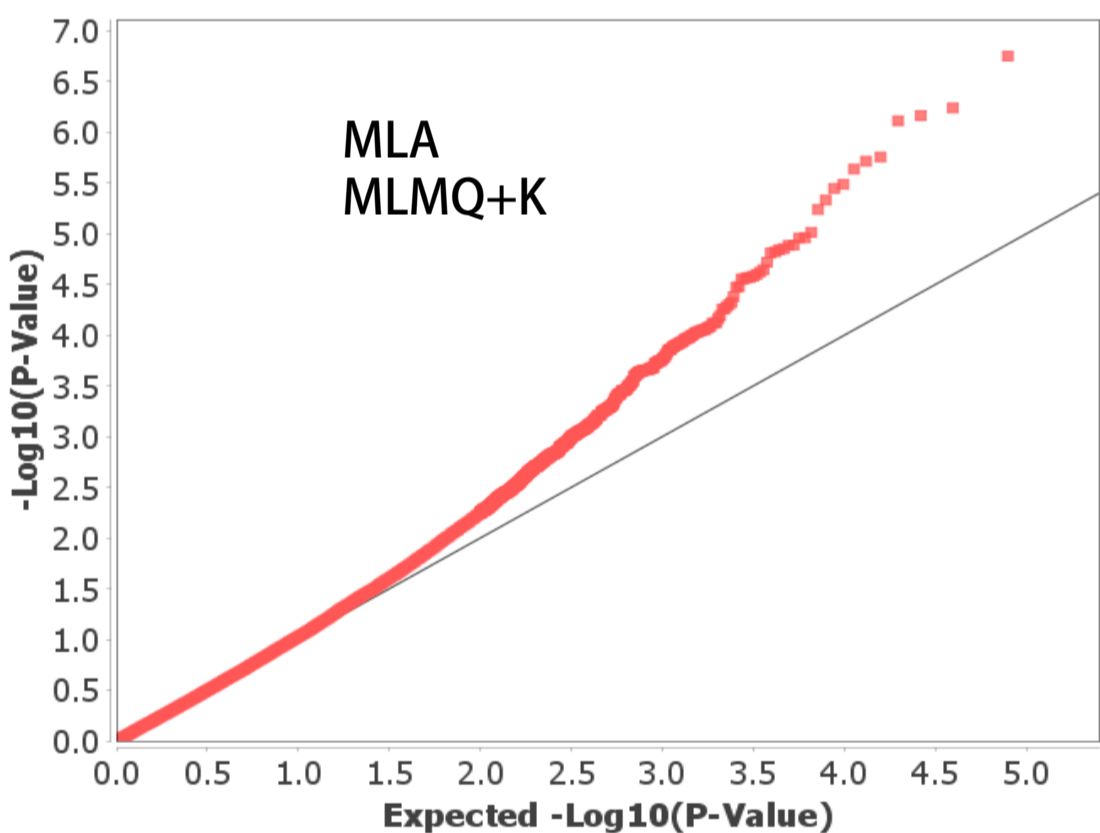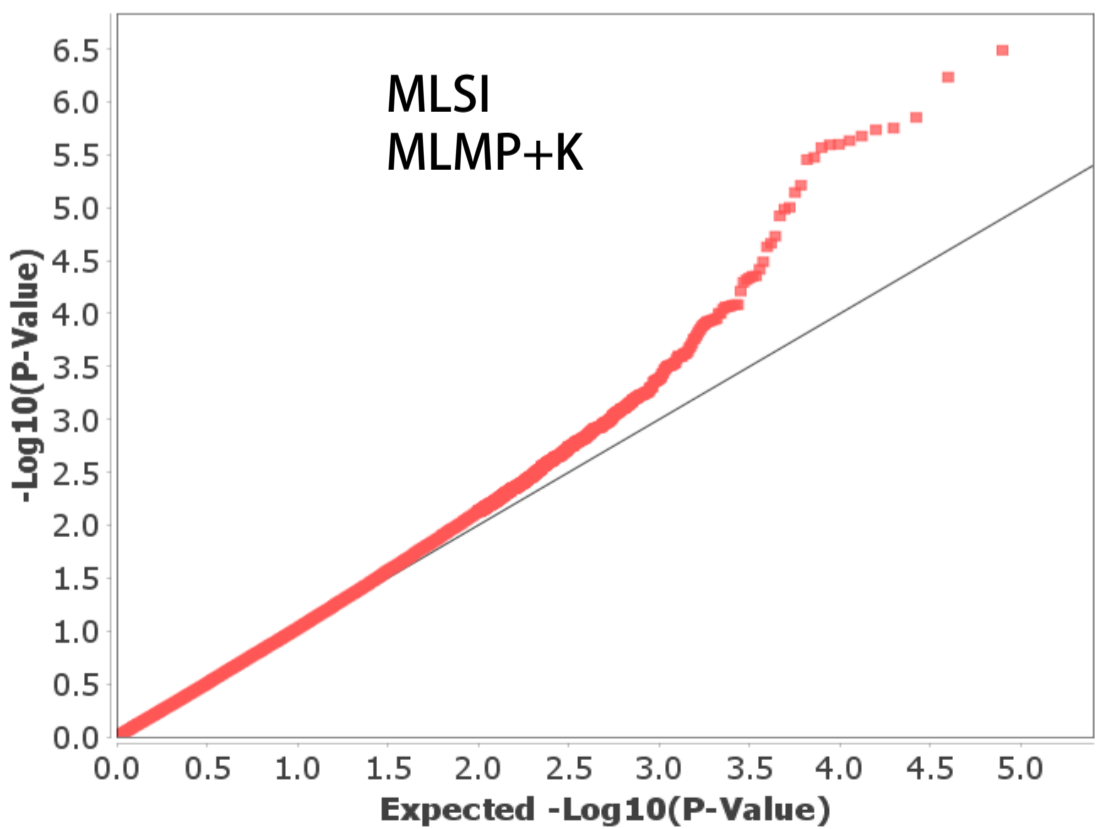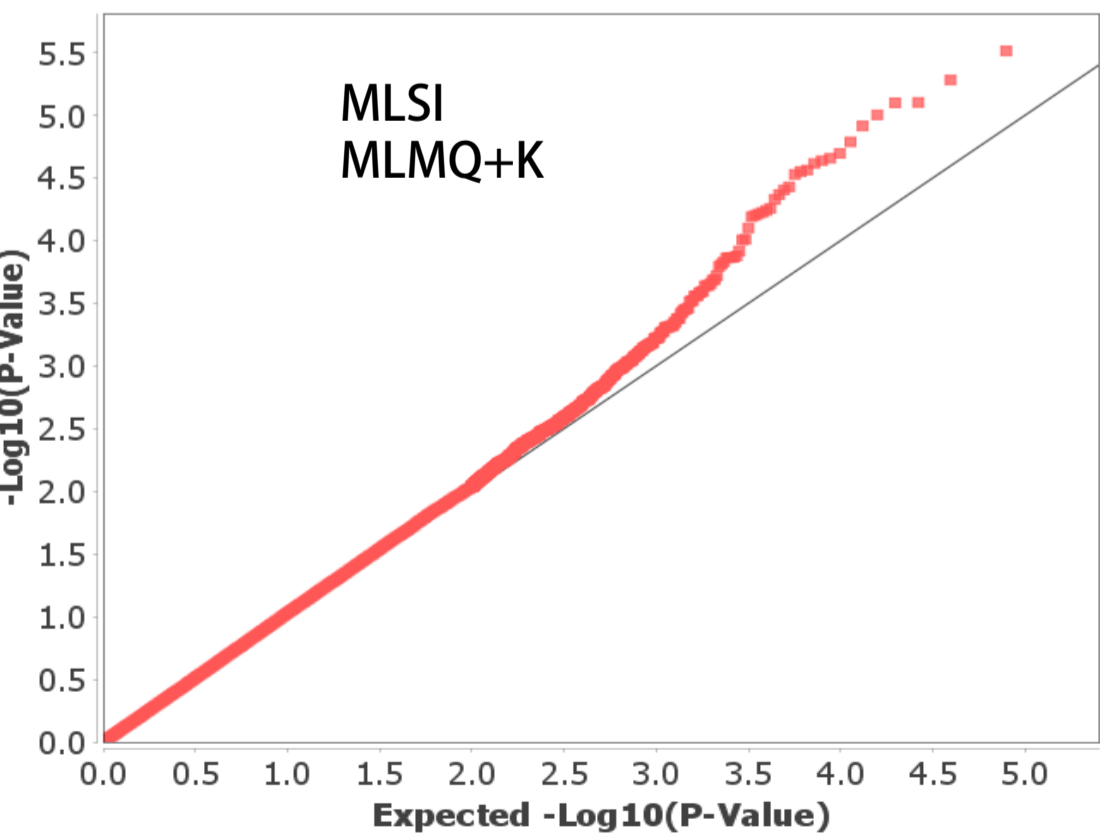

Supplement: Figure S5 — MLL: mature leaf Length, MLW: mature leaf width , MLA: mature leaf area, MLSI: mature leaf shape index. [file peerj-08-8572-s005.pdf]

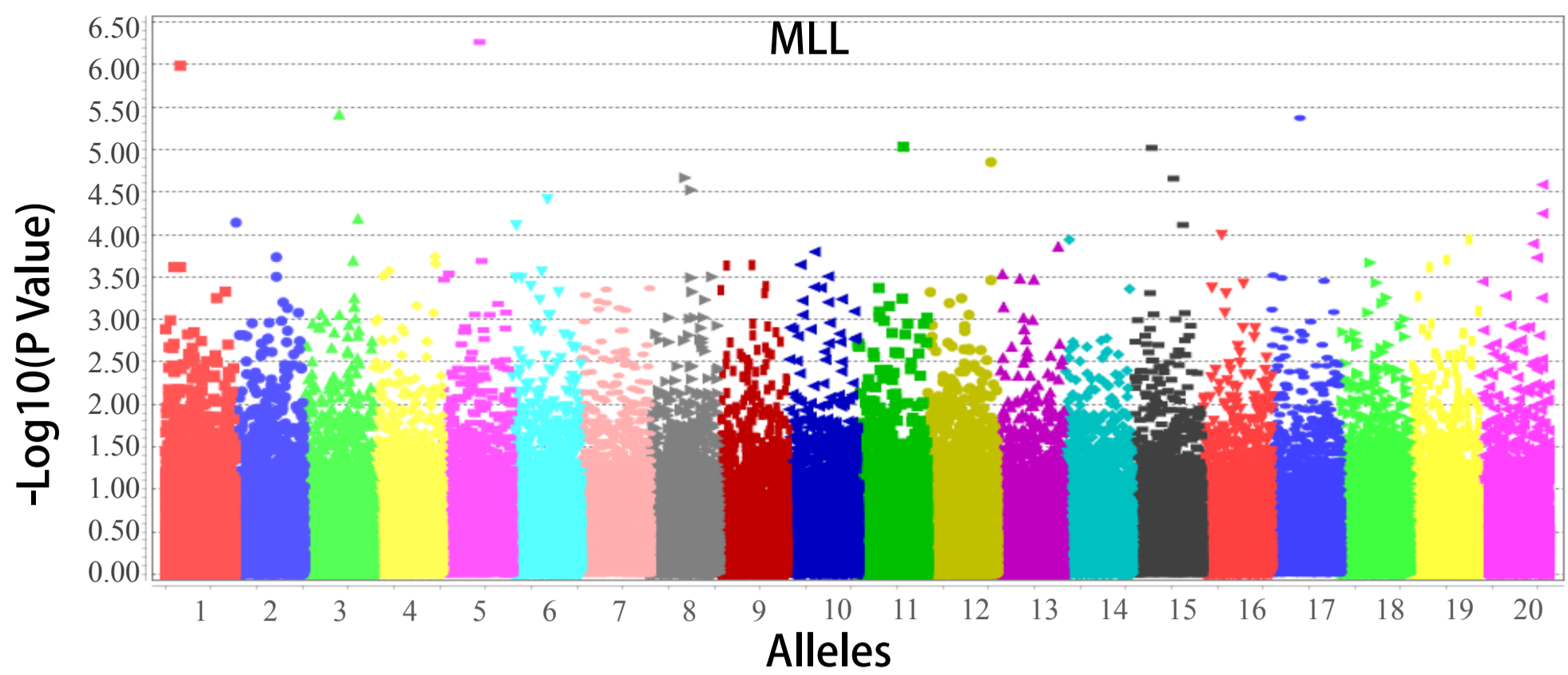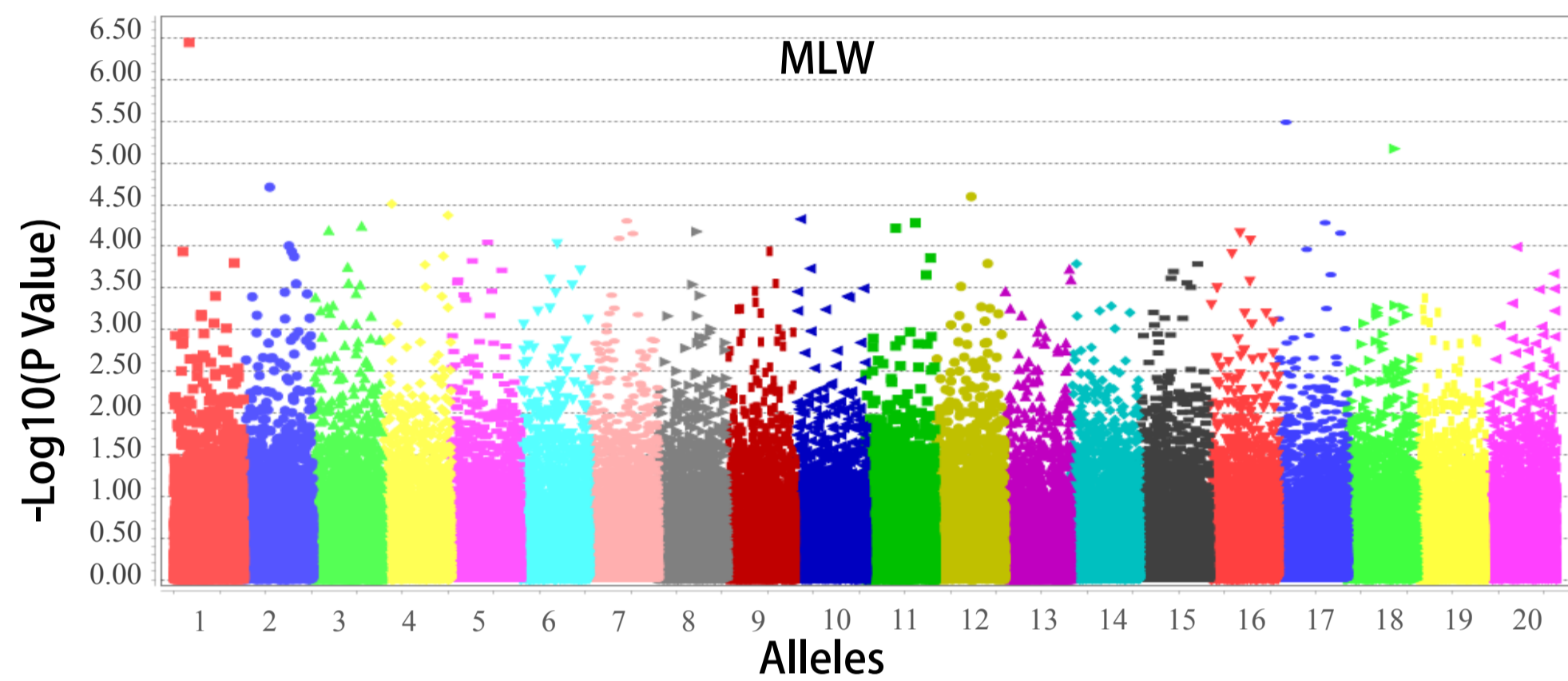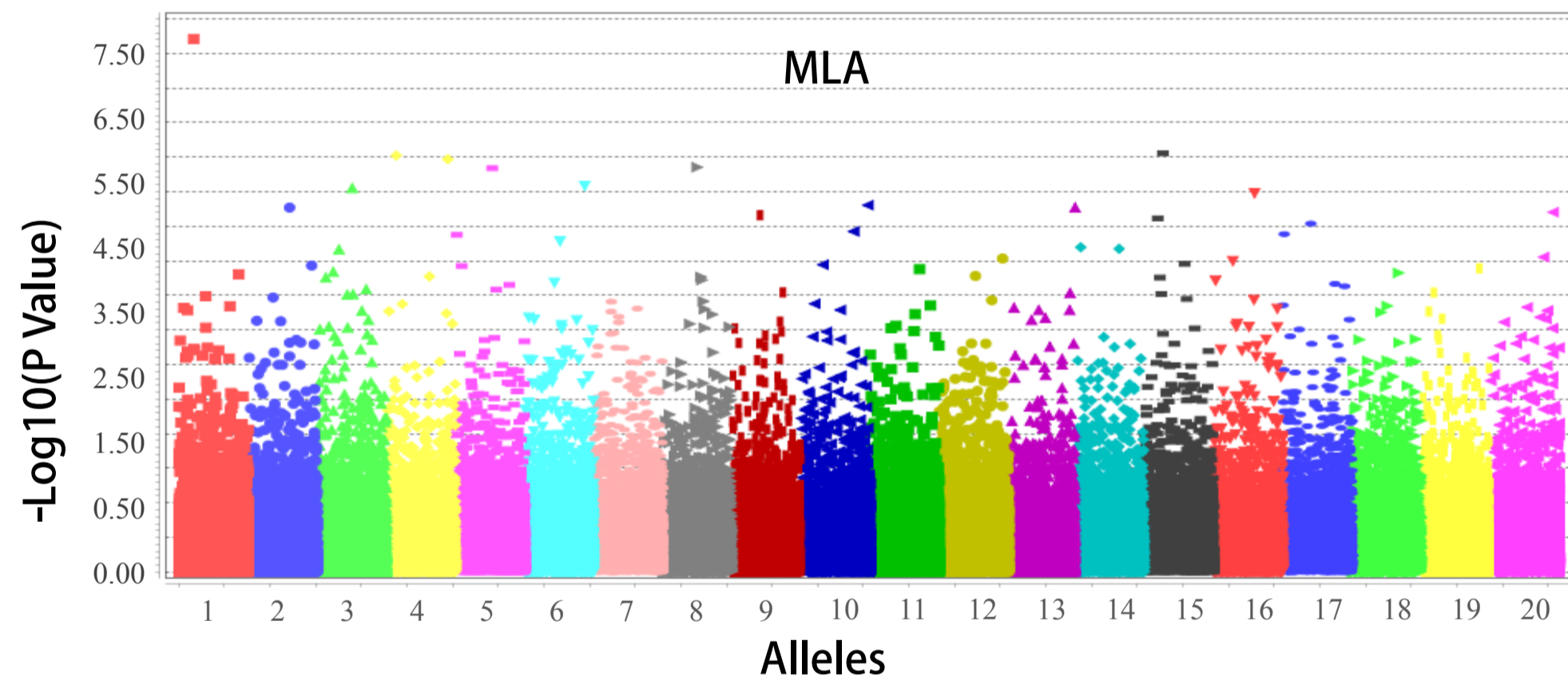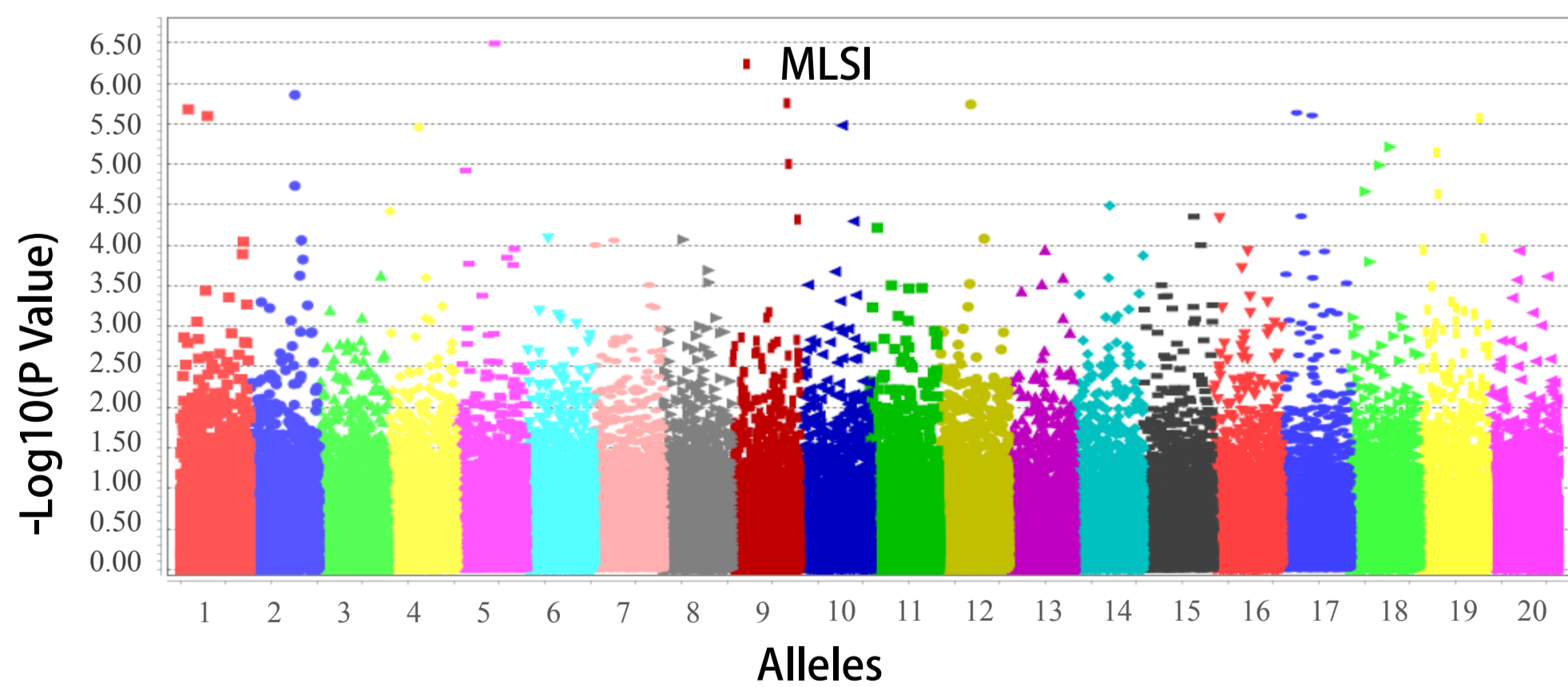

Supplement: Figure S6 [file peerj-08-8572-s006.pdf]
