# Supplementary material for: Development of core-collections for Guizhou tea genetic resources and GWAS of leaf size using SNP developed by genotyping-by-sequencing
Source: PeerJ. 2020 Mar 13;8:e8572. doi: 10.7717/peerj.8572 (PMC7075365; doi:10.7717/peerj.8572)
Supplement: Table S7 [file peerj-08-8572-s013.docx]

Table S7 SNPs significantly associated with mature leaf length (MLL), mature leaf width (MLW), mature leaf shape index (MLSI) and mature leaf area (MLA) detected by genome-wide association study (GWAS) of core-1, core-2 and mini-core

| Core collections | Trait | SNP Marker | Pse-Group | Position(bp) | Scaffold | *P*-value | *R*-square |
| --- | --- | --- | --- | --- | --- | --- | --- |
| Core-1 | MLL | PG_1:32346865 | 1 | 32346865 | Sc0000000 | 1.07E-06 | 10.11% |
|  | MLA | PG_1:32346865 | 1 | 32346865 | Sc0000000 | 4.37E-07 | 13.21% |
|  |  | PG_18:115068392 | 18 | 115068392 | xfSc0000766 | 1.73 E-06 | 10.67% |
|  |  | PG_11:34446525 | 11 | 34446525 | Sc0001907 | 1.01 E-06 | 10.79% |
|  |  | PG_12:133979444 | 12 | 133979444 | Sc0002452 | 1.83E-07 | 12.44% |
|  |  | PG_18: 35428601 | 18 | 35428601 | Sc0005070 | 1.56 E-06 | 10.15% |
|  |  | PG_13:79474508 | 13 | 79474508 | xfSc0000122 | 1.12E-06 | 11.48% |
|  | MLI | PG_1:16817455 | 1 | 16817455 | Sc0000005 | 1.19E-06 | 5.10% |
| Core-2 | MLL | PG_1:32346865 | 1 | 32346865 | Sc0000000 | 1.13E-06 | 10.70% |
|  | MLA | PG_1:32346865 | 1 | 32346865 | Sc0000000 | 3.09 E-07 | 10.39 |
|  |  | PG_12:133979444 | 12 | 133979444 | Sc0002452 | 9.18E-07 | 10.24% |
|  |  | PG_9:124262431 | 9 | 124262431 | Sc0001488 | 1.11 E-06 | 12.20% |
|  |  | PG_13:79474508 | 13 | 79474508 | xfSc0000122 | 1.32E-06 | 10.41% |
|  |  | PG_11:34446525 | 11 | 34446525 | Sc0001907 | 1.60 E-06 | 10.08% |
| Mini-core | MLA | PG_12:133979444 | 12 | 133979444 | Sc0002452 | 3.12E-07 | 13.92% |
|  |  | PG_4:10097794 | 4 | 10097794 | Sc0000326 | 6.21 E-07 | 31.30% |
|  |  | PG_13:79474508 | 13 | 79474508 | xfSc0000122 | 1.28E-06 | 15.17% |
|  |  | PG_5:120229466 | 18 | 120229466 | Sc0005395 | 1.24 E-06 | 29.26% |

Note: Pre-Group, Pseudo Group; MAF minor allele frequency.
